# Impact of Hypertension on Cancer Stage at Diagnosis Among French Women: The E3N Prospective Cohort

**DOI:** 10.1002/cam4.71021

**Published:** 2025-07-28

**Authors:** Aviane Auguste, Anna Jansana, Heinz Freisling, Pietro Ferrari, Nasser Laouali, Gianluca Severi, Marina Kvaskoff

**Affiliations:** ^1^ UVSQ, Inserm, Gustave Roussy, CESP Université Paris‐Saclay Villejuif France; ^2^ Department of Epidemiology, Biostatistics and Occupational Health, School of Population and Global Health McGill University Montréal Quebec Canada; ^3^ Nutrition and Metabolism Branch International Agency for Research on Cancer, World Health Organization Lyon France

**Keywords:** cancer, cancer screening, comorbidities, early diagnosis, France, hypertension, lung cancer, metastasis, thyroid cancer, type‐2 diabetes

## Abstract

**Introduction:**

Hypertension may delay the detection of metastatic cancers. We investigated the association between incident hypertension and the risk of metastatic onset among female cancer patients. We studied notably the role of anti‐hypertensive treatment and the time between hypertension onset and cancer diagnosis in this association.

**Methods:**

E3N is a French prospective population‐based cohort that recruited 98,995 women in 1990. A total of 7844 incident invasive cancers were examined. We used multivariate logistic regression models to calculate odds ratios (OR) and their 95% confidence intervals (CI). We also used restricted cubic splines to evaluate the nonlinear dose–response associations between hypertension duration and the risk of metastatic onset (vs. localised stage).

**Results:**

A total of 1994 cases (25%) of incident hypertension occurred before cancer diagnosis. Compared to non‐hypertensive patients, those with untreated hypertension presented more frequently with metastatic cancer among patients who regularly underwent cancer screening (OR = 1.69, 95% CI = 1.11–2.58). This association was inverse among those who did not screen regularly (OR = 0.53, 95 CI = 0.29‐0.98). Treated hypertensive patients had significantly greater odds of metastatic presentation for thyroid (OR = 2.45, 95% CI = 1.01–5.91) and lower odds for lung (OR = 0.17, 95% CI = 0.06–0.52) cancer. A significant inverse U‐shaped association with time from hypertension onset (*p* = 0.01) was observed.

**Conclusion:**

In this study, hypertension was associated with metastatic cancer presentation, but cancer screening determined the direction of the association. Time from hypertension onset was inversely associated with metastatic lung cancer, with a significant nonlinear dose–response relationship. Our findings call for further research in this area to investigate the underlying mechanisms.

**Trial Registration:**

clinicaltrials.gov identifier: NCT03285230

## Graphical abstract

Time from hypertension onset and anti‐hypertensive drugs were important determinants in the cancer stage at diagnosis in the French E3N cohort. Treated hypertension increased the odds of metastatic thyroid cancer diagnosis, whereas it lowered those odds for lung cancer. We showed that time from hypertension onset had a significant nonlinear dose–response relationship for metastatic lung cancer at diagnosis.

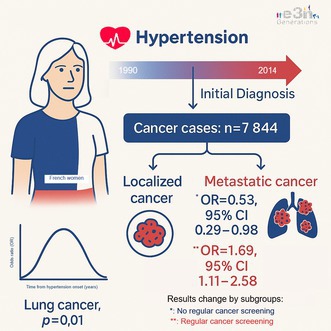

## 1 Introduction

While cancer prognosis mostly improved worldwide, progress for certain cancers and world regions has been minimal [1]. Cancer patients who are diagnosed early have greater odds of survival. In Europe, some of the most powerful tools for early cancer detection are organized population‐based screening programs for breast, colon, and cervical cancer. The efficacy of these programs is evidenced by the overall lower cancer mortality compared to countries where these programs do not exist [2]. Despite this success in Europe, significant gaps still exist as it pertains to the mortality of lung and pancreatic cancer, which make up over 50% of overall cancer mortality in France in 2022 [1, 3]. Few solutions exist to address this public health issue. Currently, low‐dose chest CT scans and blood‐based biomarkers are used as lung cancer screening tests. However, wide‐scale implementation is still inadequate as screening tests present ethical problems and high overdiagnosis rates [4, 5].

Improving early‐stage cancer diagnosis among people with multimorbidity in primary care has been recommended as a framework to avoid late‐stage cancer diagnosis [6, 7, 8]. Indeed, concurrent chronic conditions have been reported as determinants of late cancer presentation [6, 9, 10]. Hypertension, in particular, is the most frequent comorbidity among cancer patients [11]. Despite this high prevalence, the epidemiological data on hypertension and late cancer presentation are scarce [12]. The few studies that examined this association reported a significantly greater proportion of advanced thyroid cancer among patients with a personal history of hypertension [12]. In contrast, primary care visits for hypertension monitoring decreased emergency presentation for colorectal cancer in both men and women [13].

The mechanisms driving these relationships are thought to be based either on models for health‐seeking behavior, timely investigations [14, 15] or physiology [10]. To date, there is still no clear consensus on the mechanisms at play or the type of hypertensive patients most at risk for metastatic cancer. Disentangling these mechanisms and identifying targets for possible action are crucial to developing future interventions for earlier diagnosis [6, 10]. Further clarity may be achieved if we can effectively determine the severity of hypertension, antihypertension treatment, and the time interval leading to cancer diagnosis. These parameters were rarely taken into account in epidemiological studies [6]. Time intervals considered for history of hypertension by previous studies are currently limited on average to 1–3 years of cancer diagnosis [16].

In this paper, we present an analysis using data from the E3N cohort, one of the largest prospective studies in France to investigate the role of a prior history of hypertension on cancer stage at diagnosis—including the effect of time from hypertension onset. We also present the proportion of hypertension and anti‐hypertensive drug use among cancer patients since this information is also useful to determine health priorities.

## 2 Methods

### 2.1 The E3N Cohort

E3N is a large ongoing French prospective cohort of women set up in France in 1990. It comprises 98,995 women born between 1925 and 1950 from the French national health insurance plan for the national education system, the *Mutuelle Générale de l'Education Nationale* (MGEN). Women were enrolled in the cohort through a self‐administered questionnaire and were followed every 2–3 years on health and lifestyle. The average response rate to a follow‐up questionnaire was 83%, with a total loss to follow‐up of < 3% since 1990. The detailed protocol has been described elsewhere [17]. The study was approved by the French National Commission for Data Protection and Privacy; all participants gave written informed consent.

### 2.2 Validation of Cancer Cases

Cancer cases were identified mainly through self‐administered questionnaires. E3N participants were asked for their physicians' addresses and permission to contact them. Self‐reported cancers were validated based on data from pathology reports or medical records. Cancers were also identified from next‐of‐kin spontaneous reports or through information from the national death registry. Using this validation process, close to 90% of cases are histologically confirmed. Therefore, the risk of misclassification of cancer diagnosis is minimized. The cancer stage was determined using the guidelines from the UICC (*Union Internationale Contre le Cancer*) for TNM classification of malignant tumors. We considered stage I, II, and III as “localised” and stage IV as “metastatic”.

### 2.3 Study Population

Eligible E3N participants were diagnosed with an incident cancer of any site and histology between baseline and November 2014 (most recent date for validation of cases). Of the 98,995 women in the E3N cohort, there were 16,548 incident cancer cases. We excluded cases with missing stage (*n* = 3759) and in situ tumors (*n* = 1753), with no follow‐up data (*n* = 84), and those already diagnosed with hypertension at baseline (*n* = 2993). Since we were investigating the role of hypertension on late diagnosis, we also excluded cases diagnosed with hypertension less than a year prior to their cancer (*n* = 115). Participants with prevalent hypertension or cancer were excluded to reduce information bias, as these prevalent cases were not systematically verified. Additionally, participants with in situ carcinomas were excluded, as cancer‐specific mortality and disease management may differ for these patients. For our final sample, we included 7844 for analysis, among which 7348 (94%) were localized and 496 (6%) were metastatic at diagnosis.

### 2.4 Assessment of Hypertension

**FIGURE 1:**
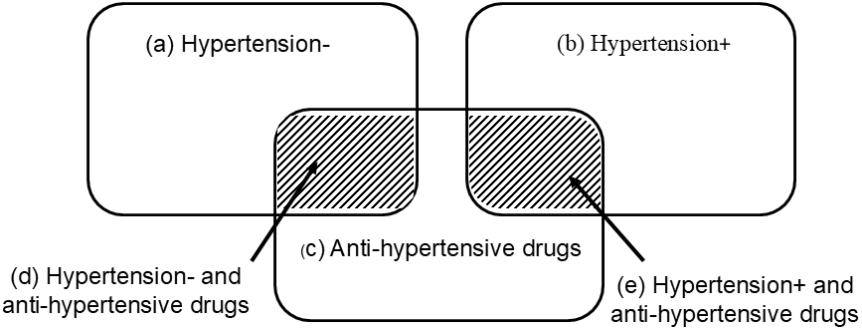
Explanation of the different categories for the hypertension variable. (a) Negative for hypertension before cancer diagnosis according to self‐report. (b) Positive for hypertension before cancer diagnosis according to self‐report. (c) Reimbursed for anti‐hypertensive drugs according to the MGEN drug reimbursement database. (d) Negative for hypertension before cancer diagnosis but reimbursed for anti‐hypertensive drugs according to self‐report. (e) Positive for hypertension before cancer diagnosis according to self‐report, and reimbursed for anti‐hypertensive drugs.

Participants reported the presence of hypertension, the date of diagnosis, and the use of anti‐hypertensive treatment in all questionnaires. In 2004, the MGEN drug reimbursement database became available for women in the study. Previously, the positive predictive value/agreement was 82% from self‐reports when cross correlating with the drug reimbursement database for anti‐hypertensive medications (diuretics, β‐blockers, calcium, and angiotensin‐converting enzyme inhibitors [Anatomical Therapeutic Chemical Classification System codes C02, C03, C07, C08, and C09, respectively]) [18]. To determine the date of diagnosis, we used self‐reported dates. For cases identified after 2004, we used either the self‐report or the first date of reimbursement for anti‐hypertensive drugs (whichever happened first). For hypertension status, study participants were divided into three groups: Absence of hypertension (HTN‐), untreated hypertension (HTN+, anti‐HTN−) and treated hypertension (HTN+, anti‐HTN+). The details and definitions are described in Figure 1.

### 2.5 Covariates

We considered measures at baseline for tobacco smoking status (former, current, and never) and education level (primary, secondary, and higher education).

We assessed total physical activity with questionnaires in 2005 that included questions on various activities. Each activity was assigned metabolic equivalents (METs) based on values from the Compendium of Physical Activities [19].

Obesity was determined based on BMI calculation using self‐reported height and weight from the baseline. BMI was determined as weight (kg) divided by squared height (m^2^). In E3N, self‐reported anthropometry has proven reliable in a validation study [20]. Participants were split into two categories: obese (BMI ≥ 30) and nonobese (BMI < 30).

We also adjusted our regression models for regular mammography, regular Pap smear and ever colonoscopy. These screening tests were measured at every questionnaire by asking “Since your last response to a questionnaire, what tests have you performed?” Screening was considered regular when tests were reported at every questionnaire (Q1‐1990 to Q11‐2014), and ever screening was considered as tests reported at least once during the follow‐up period. “No regular tests” meant that a participant was regular for neither mammography nor Pap smear and never had a colonoscopy.

Dietary data were collected in 1993 using a previously validated semi‐quantitative food frequency questionnaire including 208 food items including alcohol [21]. We used this questionnaire to build scores of adherence to the Mediterranean diet and Western diet [22]. Alcohol was measured in grams of ethanol per day. Alcohol consumption and scores for diet patterns were categorised into quartiles.

### 2.6 Statistical Analysis

We computed Clopper‐Pearson exact 95% confidence intervals (CI) to determine the uncertainty for proportions. We described the distribution of hypertension duration by treatment status and cancer stage using summary statistics. We performed Chi‐squared tests to explore potential associations between HTN status and several sociodemographic variables and screening behaviors.

The association between hypertension and cancer stage at diagnosis (metastasis vs. localised) was assessed by estimating odds ratios (ORs) and their 95% CIs using logistic regression models. Regression analyses were adjusted for age, cancer site, education level, tobacco smoking, alcohol drinking, dietary patterns (Western and Mediterranean), total physical activity, obesity, regular mammography and Pap smears, and ever colonoscopy. We attempted to adjust for incident type‐2 diabetes (T2D) as part of a sensitivity analyses. After 2004, cases were identified through the drug reimbursement insurance database. Like hypertension, the T2D validation algorithm used in the E3N cohort has been largely accepted and used in several previous publications [23, 24]. All women who were reimbursed at least twice for any glucose‐lowering medications for 1 year were considered to have validated diabetes [21]. The selection of confounders in our analyses was based on current knowledge to account for established causes of exposure and outcome without adjusting for variables on the pathway from comorbidities to stage at cancer diagnosis. The threshold for statistical significance was set to 5%. To describe the shape of the dose–response relationship between hypertension duration and the risk of metastatic cancer, we examined splines (restricted cubic spline functions with 3 knots at the 10th, 50th and 90th percentile of the distribution). Since we had fewer cases in this analysis, we had to use a minimally adjusted model to compute our results. Therefore, the restricted cubic splines were adjusted for age at cancer diagnosis, anti‐hypertensive treatment status, and cancer screening habits (regular mammography, regular pap smear, ever colonoscopy).

Missing data were observed for education level (*n* = 302), marital status (*n* = 358), tobacco smoking (*n* = 58), alcohol drinking and dietary patterns (*n* = 1449), physical activity (*n* = 1837) and obesity (*n* = 217). We used multiple imputations by chained equations (MICE) to deal with missing data [25]. The imputation model contained all the basic characteristics of the participants (age, marital status and education level), hypertension, and all adjustment variables. All variables in the imputation model that had missing values were imputed for our analyses. We generated 30 datasets. We combined the estimates and their variances/covariances into one data set using the pooling algorithm suggested by Rubin et al. to perform statistical inferences [26]. We also performed a complete case analysis on the dataset containing only observed data (Supporting Information). Statistical analyses were performed using the Statistical Analysis System software, version 9.4 (SAS Institute).

## 3 Results

### 3.1 Patient Characteristics

Table 1 shows characteristics of cases included in the final sample. Cases were on average 62 years old (Standard deviation = 8.5) at diagnosis, and the majority had a higher education degree (89%). Only 4% of cases had only primary education. In terms of cancer screening, cases screened quite frequently. Ten percent of cases had regular mammograms, and 12% had regular Pap smears. Sixty percent reported having benefitted from a colonoscopy during their lifetime. Hypertensive patients were older and had higher BMI compared to non‐hypertensive patients.

**TABLE 1:** Sociodemographic characteristics of cancer cases by hypertension status (E3N cohort, *n* = 7844).

	Total		HTN−		HTN+, anti‐HTN−		HTN+, anti‐HTN+		*p* ^a^
	*n*	%	*n*	%	*n*	%	*n*	%	
*Age*									
30–55 years	2014	(25.7)	1761	(30.1)	130	(19.3)	123	(9.3)	< 0.0001
56–65	3386	(43.2)	2484	(42.5)	301	(44.6)	601	(45.6)	
≥ 66 years	2444	(31.2)	1605	(27.4)	244	(36.2)	595	(45.1)	
*Level of education*									
Primary	278	(3.7)	195	(3.5)	26	(4.0)	57	(4.5)	< 0.0001
Secondary	559	(7.4)	395	(7.0)	58	(8.9)	106	(8.4)	
Higher education	6705	(88.9)	5032	(89.5)	571	(87.2)	1102	(87.1)	
Missing	302		228		20		54		
*Marital status*									
Single	1355	(18.1)	1034	(18.5)	108	(16.6)	213	(17.1)	0.29
Married	6131	(81.9)	4555	(81.5)	542	(83.4)	1034	(82.9)	
Missing	358		261		25		72		
*Smoking status*									
Never	3969	(51.0)	2956	(50.9)	346	(51.8)	667	(51.0)	0.56
Former	2486	(31.9)	1853	(31.9)	223	(33.4)	410	(31.4)	
Current	1331	(17.1)	1001	(17.2)	99	(14.8)	231	(17.7)	
Missing	58		40		7		11		
*Alcohol drinking*									
Q1	1489	(23.3)	1091	(23.2)	141	(24.3)	257	(23.0)	0.06
Q2	1581	(24.7)	1155	(24.6)	141	(24.3)	285	(25.5)	
Q3	1639	(25.6)	1243	(26.5)	150	(25.9)	246	(22.0)	
Q4	1686	(26.4)	1210	(25.8)	148	(25.5)	328	(29.4)	
Missing	1449		1151		95		203		
*Western diet*									
Q1	1541	(24.1)	1155	(24.6)	140	(24.1)	246	(22.0)	0.25
Q2	1584	(24.8)	1182	(25.2)	140	(24.1)	262	(23.5)	
Q3	1660	(26.0)	1209	(25.7)	144	(24.8)	307	(27.5)	
Q4	1610	(25.2)	1153	(24.5)	156	(26.9)	301	(27.0)	
Missing	1449		1151		95		203		
*Mediterranean diet*									
Q1	1620	(25.3)	1173	(25.0)	155	(26.7)	292	(26.2)	0.58
Q2	1617	(25.3)	1187	(25.3)	154	(26.6)	276	(24.7)	
Q3	1628	(25.5)	1219	(25.9)	128	(22.1)	281	(25.2)	
Q4	1530	(23.9)	1120	(23.8)	143	(24.7)	267	(23.9)	
Missing	1449		1151		95		203		
*Physical activity*									
Q1	1562	(26.0)	1127	(25.7)	118	(24.4)	317	(28.1)	0.31
Q2	1564	(26.0)	1140	(26.0)	126	(26.0)	298	(26.4)	
Q3	1506	(25.1)	1093	(24.9)	135	(27.9)	278	(24.6)	
Q4	1375	(22.9)	1033	(23.5)	105	(21.7)	237	(21.0)	
Missing	1837		1457		191		189		
*Body‐mass index*									
Underweight	310	(4.1)	265	(4.7)	15	(2.3)	30	(2.3)	< 0.0001
Normal	6223	(81.6)	4780	(83.9)	510	(78.3)	933	(72.8)	
Overweight	951	(12.5)	596	(10.5)	106	(16.3)	249	(19.4)	
Obese	143	(1.9)	54	(1.0)	20	(3.1)	69	(5.4)	
Missing	217		116		16		23		
*Mammography*									
Non regular	7066	(90.1)	5285	(90.3)	610	(90.4)	1171	(88.8)	0.22
Regular	778	(9.9)	565	(9.7)	65	(9.6)	148	(11.2)	
*Pap smear*									
Non regular	6880	(87.7)	5134	(87.8)	599	(88.7)	1147	(87.0)	0.50
Regular	964	(12.3)	716	(12.2)	76	(11.3)	172	(13.0)	
*Colonoscopy*									
Never	3134	(40.0)	2386	(40.8)	258	(38.2)	490	(37.2)	0.03
Ever	4710	(60.0)	3464	(59.2)	417	(61.8)	829	(62.9)	
*Screening status*									
No regular tests	2670	(34.0)	2043	(34.9)	222	(32.9)	405	(30.7)	0.09
One test	4137	(52.7)	3048	(52.1)	364	(53.9)	725	(55.0)	
Two tests	796	(10.1)	580	(9.9)	73	(10.8)	143	(10.8)	
Three tests	241	(3.1)	179	(3.1)	16	(2.4)	46	(3.5)	

*Note:* France, 1990–2014.

^a^ Chi‐squared test.

Table 2 shows the distribution of cancer by stage at diagnosis. We observed considerable disparities in the proportion of metastatic diagnosis by cancer type. Lung and pancreatic cancer had the highest proportion of metastatic cases (51% and 46%, respectively) whereas breast cancer had the lowest with only 1%. Head and neck and digestive cancer cases (excluding colon and pancreas) were ranked in the middle in terms of metastasis (26% and 30%, respectively).

**TABLE 2:** Proportion of hypertension and anti‐hypertensive treatment use by cancer stage (E3N cohort, *n* = 7844).

	Total		HTN+			HTN+, anti‐HTN−			HTN+, anti‐HTN+		
	*n*	%	*n*	Row%	95% CI	*n*	Row%	95% CI	*n*	Row%	95% CI
*All cancers*	7844	(100.0)	1994	25.4	(24.5–26.4)	675	8.6	(8.0–9.3)	1319	16.8	(16.0–17.7)
Localised	7348	(93.7)	1851	25.2	(24.2–26.2)	615	8.4	(7.8–9.0)	1236	16.8	(16.0–17.7)
Metastatic	496	(6.3)	143	28.8	(24.9–33.0)	60	12.1	(9.4–15.3)	83	16.7	(13.6–20.3)
*Breast*	4821	(61.5)	1145	23.8	(22.6–25.0)	373	7.7	(7.0–8.5)	772	16.0	(15.0–17.1)
Localised	4759	(98.7)	1126	23.7	(22.5–24.9)	365	7.7	(6.9–8.5)	761	16.0	(15.0–17.1)
Metastatic	62	(1.3)	19	30.6	(19.6–43.7)	8	12.9	(5.7–23.9)	11	17.7	(9.2–29.5)
*Colon*	635	(8.1)	180	28.3	(24.9–32.0)	61	9.6	(7.4–12.2)	119	18.7	(15.8–22.0)
Localised	513	(80.8)	150	29.2	(25.3–33.4)	48	9.4	(7.0–12.2)	102	19.9	(16.5–23.6)
Metastatic	122	(19.2)	30	24.6	(17.3–33.2)	13	10.7	(5.8–17.5)	17	13.9	(8.3–21.4)
*Other digestive* ^a^	121	(1.5)	32	26.4	(18.8–35.2)	14	11.6	(6.5–18.7)	18	14.9	(9.1–22.5)
Localised	85	(70.2)	21	24.7	(16.0–35.3)	11	12.9	(6.6–22.0)	10	11.8	(5.8–20.6)
Metastatic	36	(29.8)	11	30.6	(16.4–48.1)	3	8.3	(1.8–22.5)	8	22.2	(10.1–39.2)
*Gynaecological*	819	(10.4)	232	28.3	(25.3–31.6)	95	11.6	(9.5–14.0)	137	16.7	(14.2–19.5)
Localised	768	(93.8)	216	28.1	(25.0–31.5)	89	11.6	(9.4–14.1)	127	16.5	(14.0–19.4)
Metastatic	51	(6.2)	16	31.4	(19.1–45.9)	6	11.8	(4.4–23.9)	10	19.6	(9.8–33.1)
*Haematological*	12	(0.2)	2	28.6	(3.7–71.0)	0	0.0	NA	2	28.6	(3.7–71.0)
Localised	7	(58.3)	2	28.6	(3.7–71.0)	0	0.0	NA	2	28.6	(3.7–71.0)
Metastatic	5	(41.7)	0	0.0	NA	0	0.0	NA	0	0.0	NA
*Head and neck*	47	(0.6)	16	34.0	(20.9–49.3)	5	10.6	(3.6–23.1)	11	23.4	(12.3–38.0)
Localised	35	(74.5)	11	31.4	(16.9–49.3)	3	8.6	(1.8–23.1)	8	22.9	(10.4–40.1)
Metastatic	12	(25.5)	5	41.7	(15.2–72.3)	2	16.7	(2.1–48.4)	3	25.0	(5.5–57.2)
*Lung*	185	(2.4)	54	29.2	(22.8–36.3)	27	14.6	(9.8–20.5)	27	14.6	(9.8–20.5)
Localised	91	(49.2)	33	36.3	(26.4–47.0)	13	14.3	(7.8–23.2)	20	22.0	(14.0–31.9)
Metastatic	94	(50.8)	21	22.3	(14.4–32.1)	14	14.9	(8.4–23.7)	7	7.4	(3.1–14.7)
*Pancreas*	93	(1.2)	31	33.3	(23.9–43.9)	14	15.1	(8.5–24.0)	17	18.3	(11.0–27.7)
Localised	50	(53.8)	16	32.0	(19.5–46.7)	7	14.0	(5.8–26.7)	9	18.0	(8.6–31.4)
Metastatic	43	(46.2)	15	34.9	(21.0–50.9)	7	16.3	(6.8–30.7)	8	18.6	(8.4–33.4)
*Skin*	573	(7.3)	149	26.0	(22.5–29.8)	36	6.3	(4.4–8.6)	113	19.7	(16.5–23.2)
Localised	567	(99.0)	147	25.9	(22.4–29.7)	36	6.3	(4.5–8.7)	111	19.6	(16.4–23.1)
Metastatic	6	(1.0)	2	33.3	(4.3–77.7)	0	0.0	NA	2	33.3	(4.3–77.7)
*Thyroid*	337	(4.3)	81	24.0	(19.6–29.0)	19	5.6	(3.4–8.7)	62	18.4	(14.4–23.0)
Localised	292	(86.6)	67	22.9	(18.3–28.2)	17	5.8	(3.4–9.2)	50	17.1	(13.0–21.9)
Metastatic	45	(13.4)	14	31.1	(18.2–46.7)	2	4.4	(0.5–15.2)	12	26.7	(14.6–41.9)
*Urogenital*	198	(2.5)	71	35.9	(29.2–43.0)	30	15.2	(10.5–20.9)	41	20.7	(15.3–27.0)
Localised	181	(91.4)	62	34.3	(27.4–41.7)	26	14.4	(9.6–20.3)	36	19.9	(14.3–26.5)
Metastatic	17	(8.6)	9	52.9	(27.8–77.0)	4	23.5	(6.8–49.9)	5	29.4	(10.3–56.0)

*Note:* France, 1990–2014. Three participants were diagnosed with cancer of an unknown primary site.

^a^ Digestive cancers (small intestine, liver, oesophagus) excluding colon pancreas.

### 3.2 Proportion of Hypertension and Anti‐Hypertensive Treatment

**FIGURE 2:**
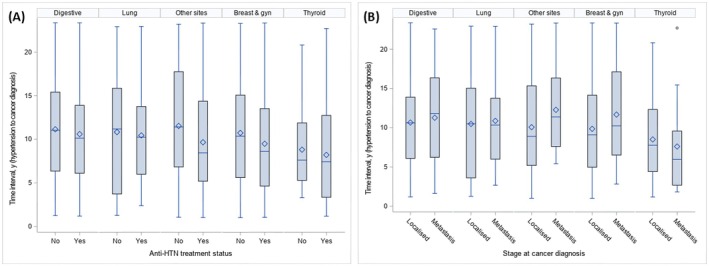
Box plot showing the distribution of time interval between hypertension and cancer diagnosis for common cancer types by (A) status for anti‐hypertensive treatment and (B) cancer stage (localised, regional, or metastatic) Box plot depicted the minimum score, the lower quartile (25%), the median (50%, horizontal line), the mean (blue diamond shape), upper quartile (75%), maximum values and outliners (black dots). Digestive cancers included the small intestine, liver, oesophagus as well as the colon and pancreas. Gyn, gynaecological cancers. E3N cohort (*n* = 7844). France, 1990–2014.

Table 2 presents the proportion of hypertension and anti‐hypertensive treatment by cancer stage. Of all cancer cases, 25.4% (CI = 24.5–26.4) developed hypertension prior to their cancer diagnosis. The proportion of untreated hypertension was higher among metastatic cases (12.1% vs. 8.4%). Figure 2 shows median time intervals between hypertension onset and cancer diagnosis. Median times were longer among untreated hypertension patients than treated (11 vs. 9 years). Compared to patients with localized cancer, median times were slightly higher for metastatic patients (10 vs. 9 years). Hypertension duration by cancer site is presented in Tables S1 and S2.

### 3.3 Association Between Hypertension and Metastasis Presentation

Table 3 displays ORs from multivariable logistic regression models assessing the association between hypertension and metastatic presentation (overall and by cancer screening behaviour). We were limited by the small sample size; therefore, we grouped certain cancers according to anatomic similarity. On one hand, hypertension was not associated with cancer stage at diagnosis among all cancer sites combined. On the other hand, significant associations were observed when we examined cancer sites separately. Compared to nonhypertensive patients, treated hypertensive patients had significantly greater odds of late presentation for thyroid cancer (OR = 2.45, 95% CI = 1.01–5.91) but lower odds for lung cancer (OR = 0.17, 95% CI = 0.06–0.52).

**TABLE 3:** Multivariate OR of cancer stage and 95% CI associated with hypertension status (E3N cohort, *n* = 7844).

	Local	Meta	Overall		Local	Meta	Non regular cancer screening		Local	Meta	Regular cancer screening^c^	
	*n*	*n*	OR^a^	95% CI	*n*	*n*	OR^b^	95% CI	*n*	*n*	OR^b^	95% CI
HTN−	5497	353	1	Ref	1877	166	1	Ref	3620	187	1	Ref
*All cancers*												
HTN+, anti‐HTN−	615	60	1.15	(0.82–1.63)	201	21	0.53	(0.29–0.98)	414	39	1.69	(1.11–2.58)
HTN+, anti‐HTN+	1236	83	0.95	(0.71–1.27)	374	31	0.87	(0.52–1.45)	862	52	1.05	(0.73–1.50)
*Breast and gynaecological*												
HTN+, anti‐HTN−	454	14	1.55	(0.87–2.79)	150	6	1.40	(0.58–3.42)	304	8	1.79	(0.81–3.92)
HTN+, anti‐HTN+	888	21	1.09	(0.66–1.81)	304	7	0.69	(0.30–1.60)	584	14	1.54	(0.81–2.95)
*Digestive* ^d^												
HTN+, anti‐HTN−	66	23	0.97	(0.55–1.69)	17	8	0.47	(0.16–1.35)	49	15	1.54	(0.81–2.94)
HTN+, anti‐HTN+	121	33	0.99	(0.61–1.58)	9	9	1.05	(0.31–3.57)	112	24	1.04	(0.61–1.76)
*Lung*												
HTN+, anti‐HTN−	13	14	0.75	(0.29–1.96)	10	5	0.15	(0.02–0.90)	3	9	4.56	(0.71–29.19)
HTN+, anti‐HTN+	20	7	0.17	(0.06–0.52)	7	4	0.13	(0.01–1.22)	13	3	0.13	(0.02–0.71)
*Thyroid*												
HTN+, anti‐HTN−	17	2	0.83	(0.16–4.38)	3	0	NA	NA	14	2	1.19	(0.20–7.26)
HTN+, anti‐HTN+	50	12	2.45	(1.01–5.91)	13	5	5.86	(0.82–41.74)	37	7	2.39	(0.75–7.64)

*Note:* France, 1990–2014. Local: localised cancer at diagnosis; Meta: metastatic cancer at diagnosis. Analyses performed using imputed data (7844 cancer cases).

^a^ Adjusted for age at cancer diagnosis, cancer site, marital status, education level, smoking status, alcohol drinking, Western diet, Mediterranean diet, physical activity, body‐mass index, regular mammography, regular pap smear and ever colonoscopy.

^b^ Adjusted for age at diagnosis, cancer site, marital status, education level, smoking status, alcohol drinking, Western diet, Mediterranean diet, physical activity and body‐mass index.

^c^ Cancer screening with at least regular mammography, regular pap smear or ever colonoscopy.

^d^ Digestive cancer includes small intestine, liver, esophagus, colon and pancreas.

Subgroup analyses by screening behavior revealed contrasting results. For all sites combined, untreated hypertensive patients presented more frequently with metastatic cancer but only among patients who regularly screened for cancer (OR = 1.69, 95% CI = 1.11–2.58). This association was inverse among those who did not screen regularly. The untreated hypertensive patients in this group had 50% lower odds of being diagnosed with a metastatic rather than localized cancer (OR = 0.53, 95% CI = 0.29–0.98). Treated hypertensive patients with lung cancer had considerably lower risk of metastatic presentation regardless of screening behavior.

### 3.4 Hypertension Duration and Metastatic Presentation

Table 4 shows the association between the time interval between hypertension onset and cancer diagnosis. The risk of metastatic presentation for lung cancer was significantly reduced only for cases recently diagnosed with hypertension (< 4 years, OR = 0.12, 95% CI = 0.02–0.70) and those with a long history of hypertension (≥ 15 years, OR = 0.19, 95% CI = 0.04–0.89). Statistical significance was maintained even after adjusting for anti‐hypertensive treatment status.

**TABLE 4:** Association between duration of hypertension and cancer stage (E3N cohort, *n* = 7844).

HTN duration (y)	Local	Meta	Model 1		Model 2	
	*n*	*n*	OR^a^	95% CI	OR^b^	95% CI
HTN‐Negative	5497	353	1	Ref	1	Ref
*All cases*						
< 4	347	21	0.97	(0.61–1.54)	1.11	(0.70–1.78)
4–14	1100	84	1.10	(0.85–1.41)	1.23	(0.94–1.61)
≥ 15	404	38	1.19	(0.83–1.71)	1.30	(0.90–1.88)
*Breast and gynaecological*						
< 4	260	3	0.58	(0.18–1.87)	0.72	(0.22–2.35)
4–14	793	22	1.34	(0.82–2.19)	1.60	(0.95–2.69)
≥ 15	289	10	1.60	(0.79–3.25)	1.85	(0.90–3.80)
*Digestive* ^c^						
< 4	21	9	1.52	(0.63–3.64)	1.66	(0.68–4.05)
4–14	124	29	0.77	(0.48–1.26)	0.85	(0.51–1.43)
≥ 15	42	18	1.29	(0.68–2.46)	1.40	(0.73–2.71)
*Lung*						
< 4	9	2	0.13	(0.02–0.75)	0.12	(0.02–0.70)
4–14	15	16	0.65	(0.26–1.61)	1.02	(0.38–2.76)
≥ 15	9	3	0.22	(0.05–0.99)	0.19	(0.04–0.89)
*Thyroid*						
< 4	15	6	4.29	(1.25–14.68)	2.36	(0.64–8.70)
4–14	45	6	1.08	(0.37–3.16)	0.80	(0.26–2.41)
≥ 15	7	2	4.14	(0.62–27.50)	2.39	(0.34–16.79)

*Note:* France, 1990–2014. Analyses performed using imputed data (7844 cancer cases).

^a^ Adjusted for age at cancer diagnosis, marital status, education level, smoking status, alcohol drinking, Western diet, Mediterranean diet, physical activity body‐mass index, regular mammography, regular pap smear, ever colonoscopy.

^b^ Model 1 with additional adjustment for anti‐hypertensive treatment status (yes/no).

^c^ Digestive cancer includes small intestine, liver, esophagus, colon and pancreas.

**FIGURE 3:**
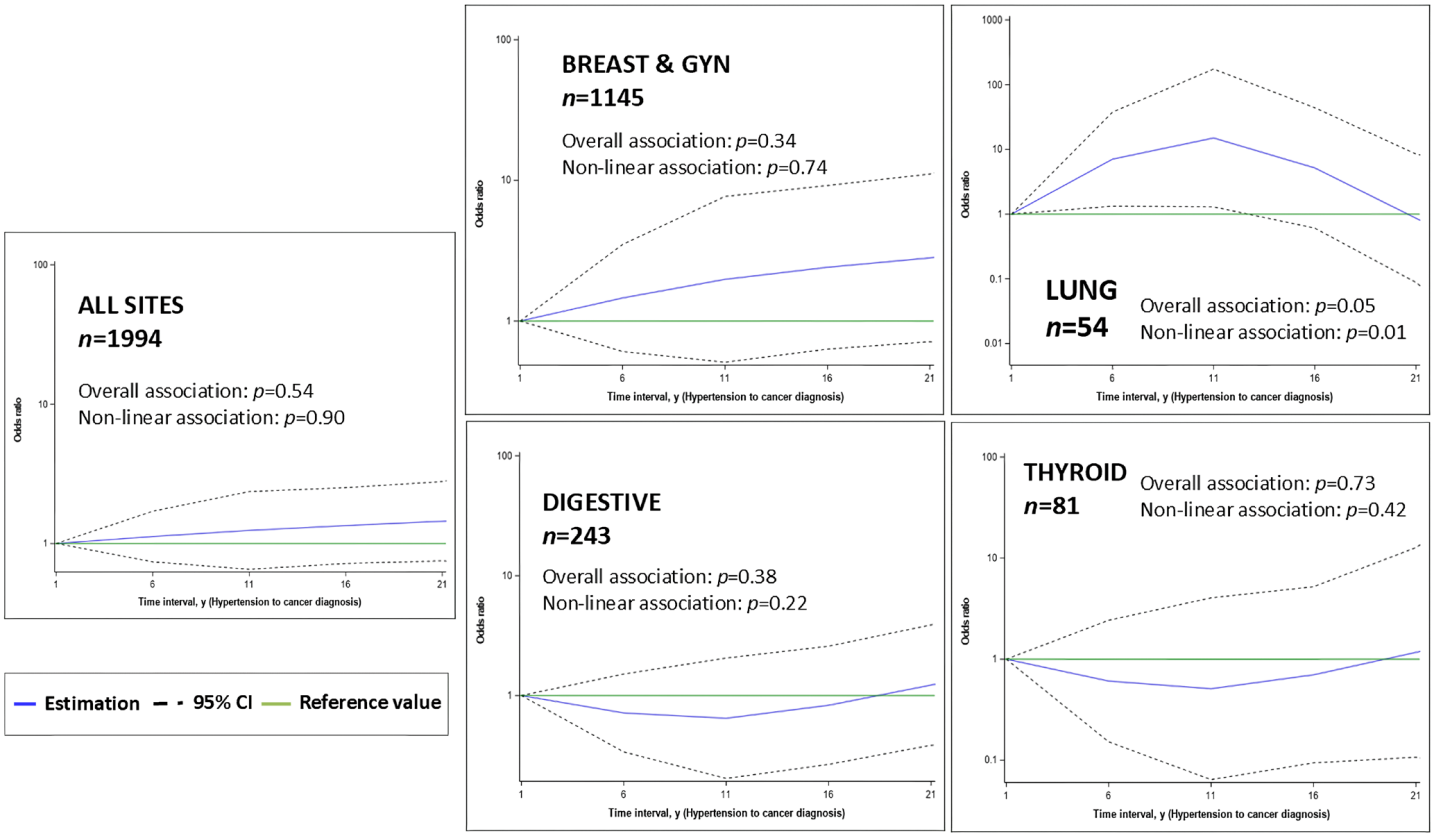
Exposure‐response associations for duration of hypertension (in years) and risk of having metastatic cancer at diagnosis. The solid blue line represents the adjusted odds ratio (OR) estimate, and the dotted black line area represents the 95% confidence interval. The reference value is defined as a hypertension duration of 1 years (solid green line). Restricted cubic splines, adjusted for age at cancer diagnosis, anti‐hypertensive treatment status and cancer screening habits (regular mammography, regular pap smear, ever colonoscopy). Analysis performed on all cancer sites overall (all sites) and stratified by cancer sites with reasonable sample size (breast and gynaecological, digestive, cancers, lung, and thyroid). E3N cohort (*n* = 7844), France, 1990–2014.

Figure 3 displays the nonlinear dose relationships between time from hypertension onset and risk of metastatic presentation. The dose–response relationship was overall linear for all cancer sites combined (OR = 1.02, 95%, CI = 1.00–1.03). We observed a U‐shaped pattern for this relationship among thyroid (Nonlinear, *p* = 0.42) and digestive cancer patients (Nonlinear *p* = 0.22). Lung cancer, in particular, revealed a significant inverted U‐shaped association between time from hypertension onset to cancer and the odds of metastasis at diagnosis. The results for the complete case analyses are presented in Tables S3 and S4.

### 3.5 Sensitivity Analysis on T2D

Multiple concurrent chronic conditions may confound the effects of hypertension on metastatic presentation. Introducing T2D into the model made no significant difference to our results for hypertension and cancer metastasis overall (data not shown). We evaluated T2D since it is often associated with hypertension. Incident T2D prior to cancer diagnosis represented only 2% of total cases (157/7844). Among those T2D cases, 53% were hypertensive (83/157). We observed a significant effect for T2D on cancer metastasis overall (data not shown: OR = 0.38, 95% CI = 0.16–0.94). We were unable to conclude on a significant role for screening behavior in this association. However, we observed that metastatic cancer presentation was less likely in both the regular (OR = 0.31, 95% CI = 0.09–1.09) and non‐regular screening groups (OR = 0.37, 95% CI = 0.09–1.46). We were not able to compute estimates for T2D across cancer sites because of too few cases.

## 4 Discussion

We conducted the largest and most comprehensive study to date on metastatic cancer presentation and hypertension. Hypertension was indeed associated with a greater risk of metastatic presentation at diagnosis overall but a lower risk for lung cancer. Hypertension affects nearly a third of cancer patients [11, 27]; therefore, the potential for cancer prevention is significant.

As shown previously, results differ by cancer site [16]. Untreated hypertension patients had suggestively higher risk of being diagnosed with breast and gynecological cancers—despite the OR not reaching statistical significance probably due to very few metastases. Albeit, our observation is consistent with a population‐based study from New Zealand which reported a significant positive association for breast cancer [16, 28]. Our results on colon cancer did not provide evidence in favor of any significant association—similarly to that same New Zealand study [16, 28]. Other studies have however reported significant associations in favor of hypertensive patients being less at risk for metastatic diagnosis for breast and colon cancer [10, 13].

We demonstrated that treated hypertensive patients were less likely to be diagnosed with metastatic lung cancer; however, the opposite was found for thyroid cancer. Our observation of a significant positive association for thyroid cancer is inconsistent with that of a single‐centre study from China which did not show any association between hypertensive patients and higher cancer stage among female patients or users of common anti‐hypertensive therapies (calcium channel blockers, angiotensin‐converting and enzyme inhibitors/angiotensin II‐receptor blockers) [12]. This incongruence may be due to selection bias owing to their single‐centre study design.

Mechanisms driving these associations have been unclear [6, 10]. Our study was able to explore some of these mechanisms by investigating screening tests and time from hypertension onset. Indeed, metastatic presentation was more common among untreated hypertension despite frequent cancer screening. This supports the hypothesis of “over‐reassurance” in which patients or physicians may not react to potential symptoms based on a recent false alarm for cancer or an “all‐clear” [6, 29]. An all‐clear diagnosis can delay help‐seeking for months or even years in case of potential cancer symptoms such as palpable breast lumps [14, 29, 30]. Our data also support the hypotheses of “alternative explanations” and “competing demands” [6, 15]. These two hypotheses suggest that cancer diagnosis is delayed because cancer symptoms are attributed to pre‐existing conditions. Competing demands with pre‐existing conditions also influence providers' decision to delay investigations or invasive procedures, notably among patients with high blood pressure [29].

Hypotheses, however, were different for thyroid and lung cancer. Lung cancer metastasis was significantly less likely among hypertensive patients—even among those who did not regularly screen for cancer. These data support a potential “surveillance effect” and/or “self‐efficacy” for prompt lung cancer diagnosis among hypertensive patients. This is plausible because in a previous study, hypertensive patients' likelihood of seeking medical attention for persistent cough (common symptom of lung cancer) was two‐fold higher compared to non‐hypertensive patients [31]. Regular screening in the E3N cohort is correlated to frequent medical visits with health care providers in general [32]. This suggests that the reduced odds for metastatic lung cancer at first presentation could be due to more opportunities for prompt referral for investigation [33], or to diagnose cancer incidentally [34]. Incidental diagnosis during primary care visits for hypertension monitoring has already been shown to favor earlier lung cancer presentation in qualitative studies [13] but we are seeing this association for the first time in a large epidemiological study [16].

Our data also support the possibility of a physiological effect resulting in a pathological interaction with carcinogenesis and hypertension and/or anti‐hypertensive drugs. We showed two instances of this in our study (thyroid and lung cancer). To date, aspirin and nonsteroidal anti‐inflammatory drugs have been suspected of significantly lowering risk late cancer presentation [10, 35]. However, evidence on the effect of anti‐hypertensive drugs is scant [36]. Nonetheless, our findings corroborate with those of a Japanese study that showed a non‐statistically significant reduction in the risk of metastasis among users of anti‐hypertensive drugs following the resection of a primary lung cancer (Hazards ratio = 0.75, 95% CI = 0.36–1.55) [37]. This association with anti‐hypertensive drugs was not assessed among thyroid cancer patients in that Japanese study. That same study demonstrated, however, a greater risk of metastasis from the use of anti‐hypertensive drugs among pancreatic, kidney, and skin cancer patients [37]. Therefore, these new data on thyroid cancer further substantiate a potential physiological effect of anti‐hypertensive drugs on metastatic cancer diagnosis.

We modelled for the first time the nonlinear dose–response relationship between the time from hypertension onset and the risk of metastasis. Overall, the risk of cancer metastasis was increased with the years from hypertension onset. Whereas the significant protective effect for metastatic presentation was present only among lung cancer patients with shorter or longer periods from hypertension onset (inverted U‐shape). We also showed some evidence of a U‐shaped association for thyroid and colon cancer. Our results therefore underscore the importance of taking into account the duration of comorbidities when it comes to assessing the risk of metastatic presentation.

We acknowledge our study limitations. E3N is indeed one of the largest and longest prospective cohorts in France; however, this is a cohort of women in the French public education service. Therefore, our findings may not be generalizable to men and the wider female population, especially since men have more inherent barriers to help‐seeking than women [38]. Also, women of a higher socioeconomic status (SES) are overrepresented in this sample compared to the general French population. Sample size and few metastatic diagnoses (*n* = 496, 6%) were another limitation that made it difficult to conclude on certain statistical associations. This was particularly low for breast cancer (1%). We know that women of higher SES, like those in the E3N cohort, detect their breast cancer at an earlier stage and have better outcomes compared to women of lower SES [39]. Therefore, metastasis in this cohort is relatively low compared to the general French population as well as the USA and Europe [10]. Nonetheless, we believe that this large French cohort provided reliable estimates for French women with a similar socioeconomic background. In addition to selection bias, we had a few issues with precision in our study. Certain subgroup analyses were constrained by low numbers, leading to wider intervals and reflecting statistical uncertainty. Therefore, these results should be interpreted with caution, albeit the key conclusions of the study are not substantially affected by this uncertainty. Multiple concurrent chronic conditions are known to affect the help‐seeking and clinical investigation process, but we did not adjust for this potential confounding effect. We tested, however, T2D as a covariable in our models, and it did not make any significant changes to our results. Despite the relatively small number of T2D cases with metastatic cancer, the T2D data were robust, validated through drug reimbursement records, and provided reliable adjustments to evaluate the robustness of the primary findings. In addition, a fair amount of data were missing for adjustment variables; however, we handled these data using multiple imputations, which produce unbiased estimates provided that the data are missing at random. Also, the reliance on self‐reported data are a limitation and a source of recall bias. However, our study's strength lies in the validation of HTN, T2D, and cancer cases. Despite the robustness of these validations within the E3N cohort, we acknowledge that the inclusion of population‐based registries would have further augmented the internal validity of our study.

The severity of comorbidities to be studied is another important factor in early diagnosis research that we did not take into account. Data on blood pressure readings is only partially available through vague self‐report measures and would not be appropriate for this study [17]. Even without disease severity, our contribution is still significant since we incorporated elements that have been crucially lacking in previous studies such as the use of anti‐hypertensive treatment and time interval from hypertension onset.

Our findings highlight several areas for consideration to improve early cancer diagnosis in France and beyond. First, untreated hypertension patients experience a greater incidence of metastatic presentation despite regular medical visits and cancer screening. Though we cannot exclude the competing demands and alternate explanation hypotheses, over‐reassurance appears to be a plausible driver for this greater risk of metastatic presentation in French women. Therefore, reliable tools are needed to inform the decision‐making process in both patients and providers to avoid missed opportunities for investigation of symptoms while minimizing overdiagnosis as is already the case for thyroid cancer in certain countries [40]. If these findings are confirmed in other studies, they would suggest a potential for ultimately reducing the risk of late cancer presentation by integrating cancer prevention and diagnostic services into existing primary care systems [7, 8]. Cancer symptom enquiry could be explicitly incorporated into routine surveillance of hypertensive patients. At last, health authorities may consider investing in the implementation of cancer decision‐support tools incorporating risk models, driven by artificial intelligence [41].

In terms of future research, we need a better understanding of the mechanisms to design and prioritize interventions targeting hypertensive patients to reduce the risk of late cancer presentation. We need to quantify the extent to which delays are influenced by help‐seeking and/or the coordination of cancer investigation. Moving forward, we also need large studies that can successfully characterize hypertension severity, duration, and treatment. Furthermore, these studies should incorporate variables for both health‐seeking behavior and clinical decisions and investigations through linkage with medico‐administrative databases.

## 5 Conclusion

Hypertension was associated with cancer stage at diagnosis among French women in the E3N cohort. Treated hypertension patients had an increased risk of metastatic thyroid cancer. We also showed for the first time a significant inverse nonlinear dose–response relationship with time from hypertension onset for metastatic lung cancer. More research is needed on mechanisms linking hypertension and late cancer presentation. However, this new evidence may suggest that a considerable reduction in metastatic cancer diagnosis is achievable by integrating cancer prevention and diagnostic services into a primary care framework. Such interventions are also feasible in low‐and‐middle‐income countries which can leverage their existing hypertension management infrastructure [7, 8].

## Author Contributions

**Aviane Auguste:** conceptualization (equal), formal analysis (lead), investigation (lead), methodology (lead), project administration (equal), validation (equal), visualization (equal), writing – original draft (lead), writing – review and editing (lead). **Anna Jansana:** investigation (supporting), methodology (supporting), validation (supporting), visualization (supporting), writing – review and editing (supporting). **Heinz Freisling:** conceptualization (lead), funding acquisition (lead), investigation (supporting), methodology (supporting), resources (lead), supervision (lead), validation (supporting), visualization (supporting), writing – review and editing (supporting). **Pietro Ferrari:** conceptualization (lead), funding acquisition (lead), validation (supporting), writing – review and editing (supporting). **Nasser Laouali:** investigation (supporting), validation (supporting), writing – review and editing (supporting). **Gianluca Severi:** conceptualization (lead), data curation (lead), funding acquisition (lead), writing – review and editing (supporting). **Marina Kvaskoff:** conceptualization (lead), data curation (lead), investigation (supporting), methodology (supporting), resources (lead), supervision (lead), validation (supporting), visualization (supporting), writing – review and editing (supporting).

## Ethics Statement

The E3N cohort was granted ethics approval by the French National Commission for Computed Data and Individual Freedom (Commission Nationale de l'Informatique et des Libertés) and all E3N participants provided written consent for participation in the study. The study was performed in accordance with the Declaration of Helsinki.

## Conflicts of Interest

The authors declare no conflicts of interest.

## Supporting information


**Table S1.** Distribution of time interval between hypertension and cancer diagnosis for common cancer types by cancer stage (E3N cohort, *n* = 7844).
**Table S2.** Distribution of time interval between hypertension and cancer diagnosis for common cancer types by status for antihypertensive treatment (E3N cohort, *n* = 7844).
**Table S3.** Association between hypertension and metastatic cancer diagnosis (complete case analysis).
**Table S4.** Association between duration of hypertension and metastasis (complete case analysis).

## Data Availability

The datasets generated and/or analysed during the current study are not publicly available since personal health data underlying the findings are protected by the French Data Protection Act. Data are however are available from the corresponding author on reasonable request.
