# Correction to “Impact of Hypertension on Cancer Stage at Diagnosis Among French Women: The E3N Prospective Cohort”

**DOI:** 10.1002/cam4.71623

**Published:** 2026-02-12

A. Auguste, A. Jansana, H. Freisling, et al., “Impact of Hypertension on Cancer Stage at Diagnosis Among French Women: The E3N Prospective Cohort,” *Cancer Medicine* 14, no. 15 (2025): e71021, https://doi.org/10.1002/cam4.71021.

In the Acknowledgements section, a Disclaimer is missing. The following text should be added: “Where authors are identified as personnel of the International Agency for Research on Cancer or WHO, the authors alone are responsible for the views expressed in this Article and they do not necessarily represent the decisions, policy, or views of the International Agency for Research on Cancer or WHO.”

We apologize for this error.

