# Supplementary material for: Impact of Hypertension on Cancer Stage at Diagnosis Among French Women: The E3N Prospective Cohort
Source: Cancer Med. 2025 Jul 28;14(15):e71021. doi: 10.1002/cam4.71021 (PMC12302036; doi:10.1002/cam4.71021)
Supplement: Supplementary file 1 — Table S1. Distribution of time interval between hypertension and cancer diagnosis for common cancer types by cancer stage (E3N cohort, n = 7844). Table S2. Distribution of time interval between hypertension and cancer diagnosis for common cancer types by status for antihypertensive treatment (E3N cohort, n = 7844). Table S3. Association between hypertension and metastatic cancer diagnosis (complete case analysis). Table S4. Association between duration of hypertension and metastasis (complete case analysis). [file CAM4-14-e71021-s001.docx]

**SUPPLEMENTARY MATERIALS**

**Title:** Impact of hypertension on cancer stage at diagnosis among French women: the E3N prospective cohort

**Authors**

Aviane Auguste^*^, Anna Jansana, Heinz Freisling, Pietro Ferrari, Nasser Laouali, Gianluca Severi, Marina Kvaskoff

**Table S1**: Distribution of time interval between hypertension and cancer diagnosis for common cancer types by cancer stage (E3N cohort, *n*=7,844).

**Table S2**: Distribution of time interval between hypertension and cancer diagnosis for common cancer types by status for anti-hypertensive treatment (E3N cohort, *n*=7,844).

**Table S3:** Association between hypertension and metastatic cancer diagnosis (Complete case analysis).

**Table S4:** Association between duration of hypertension and metastasis (Complete case analysis).

**Table S1**: Distribution of time interval between hypertension and cancer diagnosis for common cancer types by cancer stage (E3N cohort, *n*=7,844)

|  | N obs | Minimum | Q1 | Median | Q3 | Maximum |
| --- | --- | --- | --- | --- | --- | --- |
| **All cancers** | **1994** | **1.0** | **5.1** | **9.3** | **14.4** | **23.4** |
| Localised | 1851 | 1.0 | 5.0 | 9.3 | 14.2 | 23.4 |
| Metastasis | 143 | 1.6 | 6.1 | 10.2 | 15.5 | 23.3 |
| **Head and neck** | **16** | **1.7** | **7.5** | **10.6** | **14.4** | **22.4** |
| Localised | 11 | 2 | 7 | 9 | 13 | 22 |
| Metastasis | 5 | 7 | 8 | 14 | 15 | 18 |
| **Breast** | **1145** | **1.0** | **5.0** | **9.0** | **14.3** | **23.4** |
| Localised | 1126 | 1 | 5 | 9 | 14 | 23 |
| Metastasis | 19 | 3 | 6 | 9 | 17 | 23 |
| **Colon** | **180** | **1.2** | **6.2** | **9.9** | **13.4** | **23.4** |
| Localised | 150 | 1 | 6 | 10 | 13 | 23 |
| Metastasis | 30 | 2 | 6 | 9 | 16 | 23 |
| **Other digestive** | **32** | **1.7** | **5.8** | **10.8** | **15.9** | **20.9** |
| Localised | 21 | 2 | 6 | 11 | 15 | 20 |
| Metastasis | 11 | 2 | 4 | 9 | 20 | 21 |
| **Gynaecological** | **232** | **1.3** | **5.5** | **9.5** | **14.0** | **23.1** |
| Localised | 216 | 1 | 6 | 9 | 14 | 23 |
| Metastasis | 16 | 3 | 9 | 11 | 17 | 23 |
| **Haematological** | **2** | **6.4** | **6.4** | **10.6** | **14.9** | **14.9** |
| Localised | 2 | 6 | 6 | 11 | 15 | 15 |
| Metastasis | NA | NA | NA | NA | NA | NA |
| **Lung** | **54** | **1.3** | **5.8** | **10.4** | **14.4** | **23.0** |
| Localised | 33 | 1 | 4 | 10 | 15 | 23 |
| Metastasis | 21 | 3 | 6 | 10 | 14 | 23 |
| **Pancreas** | **31** | **1.9** | **6.1** | **13.5** | **17.1** | **23.4** |
| Localised | 16 | 2 | 5 | 12 | 20 | 23 |
| Metastasis | 15 | 2 | 7 | 14 | 17 | 20 |
| **Skin** | **149** | **1.0** | **4.7** | **8.4** | **14.9** | **23.2** |
| Localised | 147 | 1 | 5 | 8 | 15 | 23 |
| Metastasis | 2 | 11 | 11 | 11 | 11 | 11 |
| **Thyroid** | **81** | **1.2** | **3.8** | **7.5** | **11.9** | **22.7** |
| Localised | 67 | 1 | 4 | 8 | 12 | 21 |
| Metastasis | 14 | 2 | 3 | 6 | 10 | 23 |
| **Urogenital** | **71** | **1.1** | **6.6** | **10.7** | **16.6** | **23.3** |
| Localised | 62 | 1 | 7 | 10 | 16 | 23 |
| Metastasis | 9 | 5 | 7 | 11 | 17 | 23 |

France. 1990–2014

**Table S2**: Distribution of time interval between hypertension and cancer diagnosis for common cancer types by status for anti-hypertensive treatment (E3N cohort, *n*=7,844)

|  | N obs | Minimum | Q1 | Median | Q3 | Maximum |
| --- | --- | --- | --- | --- | --- | --- |
| **All cancers** | **1994** | **1.0** | **5.1** | **9.3** | **14.4** | **23.4** |
| No | 675 | 1.0 | 5.6 | 10.5 | 15.4 | 23.4 |
| Yes | 1319 | 1.0 | 4.9 | 8.7 | 13.7 | 23.4 |
| **Head and neck** | **16** | **1.7** | **7.5** | **10.6** | **14.4** | **22.4** |
| No | 5 | 1.7 | 6.8 | 7.3 | 17.8 | 20.0 |
| Yes | 11 | 3.8 | 8.0 | 11.8 | 13.9 | 22.4 |
| **Breast** | **1145** | **1.0** | **5.0** | **9.0** | **14.3** | **23.4** |
| No | 373 | 1.0 | 5.7 | 10.6 | 15.6 | 23.3 |
| Yes | 772 | 1.0 | 4.4 | 8.5 | 13.1 | 23.4 |
| **Colon** | **180** | **1.2** | **6.2** | **9.9** | **13.4** | **23.4** |
| No | 61 | 1.2 | 5.6 | 10.9 | 13.3 | 23.4 |
| Yes | 119 | 1.2 | 6.4 | 9.5 | 13.5 | 23.3 |
| **Other digestive** | **32** | **1.7** | **5.8** | **10.8** | **15.9** | **20.9** |
| No | 14 | 1.9 | 8.1 | 10.9 | 15.4 | 20.1 |
| Yes | 18 | 1.7 | 4.1 | 9.4 | 16.3 | 20.9 |
| **Gynaecological** | **232** | **1.3** | **5.5** | **9.5** | **14.0** | **23.1** |
| No | 95 | 1.3 | 5.1 | 9.1 | 13.7 | 23.1 |
| Yes | 137 | 1.4 | 6.1 | 9.7 | 14.5 | 21.9 |
| **Haematological** | **2** | **6.4** | **6.4** | **10.6** | **14.9** | **14.9** |
| No | NA | NA | NA | NA | NA | NA |
| Yes | 2 | 6.4 | 6.4 | 10.6 | 14.9 | 14.9 |
| **Lung** | **54** | **1.3** | **5.8** | **10.4** | **14.4** | **23.0** |
| No | 27 | 1.3 | 3.7 | 11.2 | 15.9 | 22.9 |
| Yes | 27 | 2.4 | 6.0 | 10.2 | 13.8 | 23.0 |
| **Pancreas** | **31** | **1.9** | **6.1** | **13.5** | **17.1** | **23.4** |
| No | 14 | 2.0 | 4.6 | 14.4 | 18.2 | 22.4 |
| Yes | 17 | 1.9 | 10.1 | 13.3 | 15.8 | 23.4 |
| **Skin** | **149** | **1.0** | **4.7** | **8.4** | **14.9** | **23.2** |
| No | 36 | 1.1 | 5.3 | 11.6 | 18.5 | 23.2 |
| Yes | 113 | 1.0 | 4.5 | 7.9 | 12.6 | 22.1 |
| **Thyroid** | **81** | **1.2** | **3.8** | **7.5** | **11.9** | **22.7** |
| No | 19 | 3.3 | 5.3 | 7.6 | 11.9 | 20.8 |
| Yes | 62 | 1.2 | 3.3 | 7.4 | 12.7 | 22.7 |
| **Urogenital** | **71** | **1.1** | **6.6** | **10.7** | **16.6** | **23.3** |
| No | 30 | 1.5 | 7.1 | 10.2 | 16.6 | 21.3 |
| Yes | 41 | 1.1 | 6.6 | 10.7 | 16.3 | 23.3 |

France, 1990–2014

**Table S3:** Association between hypertension and metastatic cancer diagnosis (Complete case analysis)

|  | Local | Meta |  |  |  | Local | Meta | Non regular cancer screening | |  | Local | Meta | Regular cancer screening^c^ | |
| --- | --- | --- | --- | --- | --- | --- | --- | --- | --- | --- | --- | --- | --- | --- |
|  | *n* | *n* | OR^a^ | CI 95% |  | *n* | *n* | OR^b^ | CI 95% |  | *n* | *n* | OR^b^ | CI 95% |
| HTN- | 3389 | 153 | 1 | ref |  | 954 | 58 | 1 | ref |  | 2435 | 95 | 1 | ref |
| **All cancers** |  |  |  |  |  |  |  |  |  |  |  |  |  |  |
| HTN+, anti-HTN- | 396 | 15 | 0.76 | (0.42-1.36) |  | 101 | 2 | 0.21 | (0.05-1.00) |  | 295 | 13 | 0.96 | (0.47-1.98) |
| HTN+, anti-HTN+ | 858 | 54 | 1.18 | (0.83-1.70) |  | 237 | 20 | 1.11 | (0.59-2.12) |  | 621 | 34 | 1.12 | (0.69-1.84) |
| **Breast and gynaecological** |  |  |  |  |  |  |  |  |  |  |  |  |  |  |
| HTN+, anti-HTN- | 289 | 2 | 0.41 | (0.10-1.74) |  | 79 | 1 | 0.47 | (0.06-3.72) |  | 210 | 1 | 0.37 | (0.05-2.84) |
| HTN+, anti-HTN+ | 611 | 13 | 1.04 | (0.53-2.01) |  | 188 | 4 | 0.62 | (0.20-1.94) |  | 423 | 9 | 1.60 | (0.68-3.76) |
| **Digestive*** |  |  |  |  |  |  |  |  |  |  |  |  |  |  |
| HTN+, anti-HTN- | 39 | 5 | 0.52 | (0.19-1.44) |  | 3 | 1 | NA | NA |  | 36 | 4 | 0.57 | (0.19-1.74) |
| HTN+, anti-HTN+ | 87 | 19 | 1.00 | (0.53-1.89) |  | 7 | 4 | NA | NA |  | 80 | 15 | 1.02 | (0.52-2.03) |
| **Lung** |  |  |  |  |  |  |  |  |  |  |  |  |  |  |
| HTN+, anti-HTN- | 5 | 3 | 0.41 | (0.06-2.82) |  | 4 | 0 | NA | NA |  | 1 | 3 | 16.02 | (0.12->999) |
| HTN+, anti-HTN+ | 12 | 5 | 0.17 | (0.03-0.85) |  | 5 | 3 | NA | NA |  | 7 | 2 | 0.03 | (<0.001-0.82) |
| **Thyroid** |  |  |  |  |  |  |  |  |  |  |  |  |  |  |
| HTN+, anti-HTN- | 16 | 2 | 1.34 | (0.20-8.95) |  | 3 | 0 | NA | NA |  | 13 | 2 | 1.96 | (0.26-14.56) |
| HTN+, anti-HTN+ | 40 | 10 | 5.12 | (1.64-15.99) |  | 9 | 4 | NA | NA |  | 31 | 6 | 4.46 | (1.05-19.00) |

E3N cohort: France, 1990–2014

Complete case analysis: Analyses performed on observed data (*n*=4865).

a: Adjusted for age at cancer diagnosis, cancer site, marital status, education level, smoking status, alcohol drinking, Western diet, Mediterranean diet, physical activity, body-mass index, regular mammography, regular pap smear and ever colonoscopy.

b: Adjusted for age at diagnosis, cancer site, marital status, education level, smoking status, alcohol drinking, Western diet, Mediterranean diet, physical activity and body-mass index.

c: Cancer screening with at least regular mammography, regular pap smear or ever colonoscopy

Anti-HTN tt: Anti-hypertensive treatment.

*: Digestive including colon pancreas

Local: Localised cancer at diagnosis, Meta: Metastatic cancer at diagnosis

**Table S4:** Association between duration of hypertension and metastasis (Complete case analysis)

| HTN duration (y) | Local | Meta | Model 1 | |  | Model 2 | |
| --- | --- | --- | --- | --- | --- | --- | --- |
|  | *n* | *n* | OR^a^ | CI 95% |  | OR^b^ | CI 95% |
| HTN-Negative | 3389 | 153 | 1 | ref |  | 1 | ref |
| **All cases** |  |  |  |  |  |  |  |
| <4 | 219 | 10 | 1.32 | (0.65-2.68) |  | 1.22 | (0.59-2.54) |
| 4-14 | 732 | 35 | 0.83 | (0.55-1.26) |  | 0.78 | (0.50-1.22) |
| >=15 | 303 | 24 | 1.42 | (0.85-2.37) |  | 1.36 | (0.81-2.30) |
| **Breast and gynaecological** |  |  |  |  |  |  |  |
| <4 | 168 | 2 | 0.70 | (0.16-3.01) |  | 0.69 | (0.15-3.07) |
| 4-14 | 513 | 7 | 0.69 | (0.30-1.60) |  | 0.68 | (0.28-1.65) |
| >=15 | 219 | 6 | 1.40 | (0.56-3.52) |  | 1.38 | (0.53-3.59) |
| **Digestive** |  |  |  |  |  |  |  |
| <4 | 9 | 3 | 2.14 | (0.48-9.52) |  | 2.38 | (0.52-11.02) |
| 4-14 | 91 | 10 | 0.44 | (0.20-0.96) |  | 0.50 | (0.22-1.16) |
| >=15 | 26 | 11 | 1.45 | (0.63-3.36) |  | 1.56 | (0.66-3.70) |
| **Lung** |  |  |  |  |  |  |  |
| <4 | 2 | 0 | NA |  |  | NA |  |
| 4-14 | 8 | 6 | 0.34 | (0.06-1.77) |  | 0.31 | (0.05-1.91) |
| >=15 | 7 | 2 | 0.16 | (0.02-1.45) |  | 0.16 | (0.02-1.46) |
| **Thyroid** |  |  |  |  |  |  |  |
| <4 | 12 | 5 | 9.96 | (1.92-51.67) |  | 5.34 | (0.93-30.53) |
| 4-14 | 38 | 6 | 2.28 | (0.63-8.30) |  | 1.25 | (0.30-5.16) |
| >=15 | 6 | 1 | 4.01 | (0.29-54.95) |  | 2.49 | (0.16-37.69) |

E3N cohort: France, 1990–2014

Complete case analysis: Analyses performed on observed data (*n*=4865).

a: Adjusted for age at cancer diagnosis, cancer site, marital status, education level, smoking status, alcohol drinking, Western diet, Mediterranean diet, physical activity body-mass index, regular mammography, regular pap smear, ever colonoscopy.

b: Model 1 with additional adjustment for anti-hypertensive treatment status (yes/no)

*: Digestive cancer includes small intestine, liver, oesophagus, colon and pancreas.
